# Alternative splicing variations in mouse CAPS2: differential expression and functional properties of splicing variants

**DOI:** 10.1186/1471-2202-8-25

**Published:** 2007-04-12

**Authors:** Tetsushi Sadakata, Miwa Washida, Teiichi Furuichi

**Affiliations:** 1 Laboratory for Molecular Neurogenesis, RIKEN Brain Science Institute, 2-1 Hirosawa, Wako, Saitama 351-0198, Japan

## Abstract

**Background:**

Ca^2+^-dependent activator protein 2 (CAPS2/CADPS2) is a secretory vesicle-associated protein involved in the release of neurotrophin. We recently reported that an aberrant, alternatively spliced CAPS2 mRNA that lacks exon 3 (CAPS2Δexon3) is detected in some patients with autism. Splicing variations in mouse CAPS2 and their expression and functions remain unclear.

**Results:**

In this study, we defined 31 exons in the mouse CAPS2 gene and identified six alternative splicing variants, CAPS2a-f. CAPS2a is an isoform lacking exons 22 and 25, which encode part of the Munc13-1-homologous domain (MHD). CAPS2b lacks exon 25. CAPS2c lacks exons 11 and 22. CAPS2d, 2e, and 2f have C-terminal deletions from exon 14, exon 12, and exon 5, respectively. On the other hand, a mouse counterpart of CAPS2Δexon3 was not detected in the mouse tissues tested. CAPS2b was expressed exclusively in the brain, and the other isoforms were highly expressed in the brain, but also in some non-neural tissues. In the brain, all isoforms showed predominant expression patterns in the cerebellum. In the developing cerebellum, CAPS2b showed an up-regulated expression pattern, whereas the other isoforms exhibited transiently peaked expression patterns. CAPS2 proteins were mostly recovered in soluble fractions, but some were present in membrane fractions, except for CAPS2c and 2f, both of which lack the PH domain, suggesting that the PH domain is important for membrane association. In contrast to CAPS2a and 2b, CAPS2c showed slightly decreased BDNF-releasing activity, which is likely due to the C-terminal truncation of the PH domain in CAPS2c.

**Conclusion:**

This study indicates that, in mouse, there are six splicing variants of CAPS2 (CAPS2a-f), and that these are subdivided into two groups: a long form containing the C-terminal MHD and a short form lacking the C-terminal MHD. These results demonstrate that the splicing variations correlate with their expression patterns and intracellular distribution, and affect BDNF release; however, whether or not the short forms possess activities other than BDNF release, for example as natural dominant-negative isoforms, remains to be determined.

## Background

The Ca^2+^-dependent activator protein for secretion (CAPS/CADPS) family consists of two members, CAPS1/CADPS1 [1-3] and CAPS2/CADPS2 [4-6]. CAPS1 was shown to play a role in the ATP-dependent priming step of Ca^2+^-triggered dense-core vesicle (DCV) exocytosis by binding to both phosphatidyl inositol 4,5-bisphosphate and DCVs in response to Ca^2+ ^increase [7,8]; it also interacts with the dopamine receptor [9]. A study of CAPS1-deficient mice suggested that CAPS1 is involved in catecholamine loading of DCVs in embryonic chromaffin cells [10]. On the other hand, CAPS2 is associated with secretory vesicles containing the neurotrophins BDNF and NT-3 in the parallel fiber terminals of cerebellar granule cells, and is involved in the release of BDNF and NT-3 [6]. CAPS2 is widely localized across various brain regions, and shows overlapping distribution patterns with BDNF in many areas [11]. Our recent studies of CAPS2-deficient mice indicated that CAPS2 plays pivotal roles in BDNF release, cellular phenotypes (e.g., neuronal survival and differentiation, synapse structure and function), and behavioral phenotypes (e.g., water-maze spatial learning, anxiety, circadian rhythm, maternal behavior) [12,13].

The expression of an aberrant splicing variant of CAPS2 in some patients with autism has also been reported [12]. The autistic isoform, CAPS2Δexon3, is missing exon 3, which encodes the dynactin 1-interacting domain (DID) [12]. Exogenously expressed CAPS2Δexon3 failed to localize to axons but accumulated in somato-dendritic areas in cultured cerebellar and cortical neurons, suggesting a possible disturbance of local CAPS2-mediated neurotrophin release [12]. In this study, we further explore splicing variations in CAPS2 and identified five additional splice isoforms. We characterized the expression patterns and neurotrophin release properties of these isoforms.

## Results and discussion

**Figure 1:**
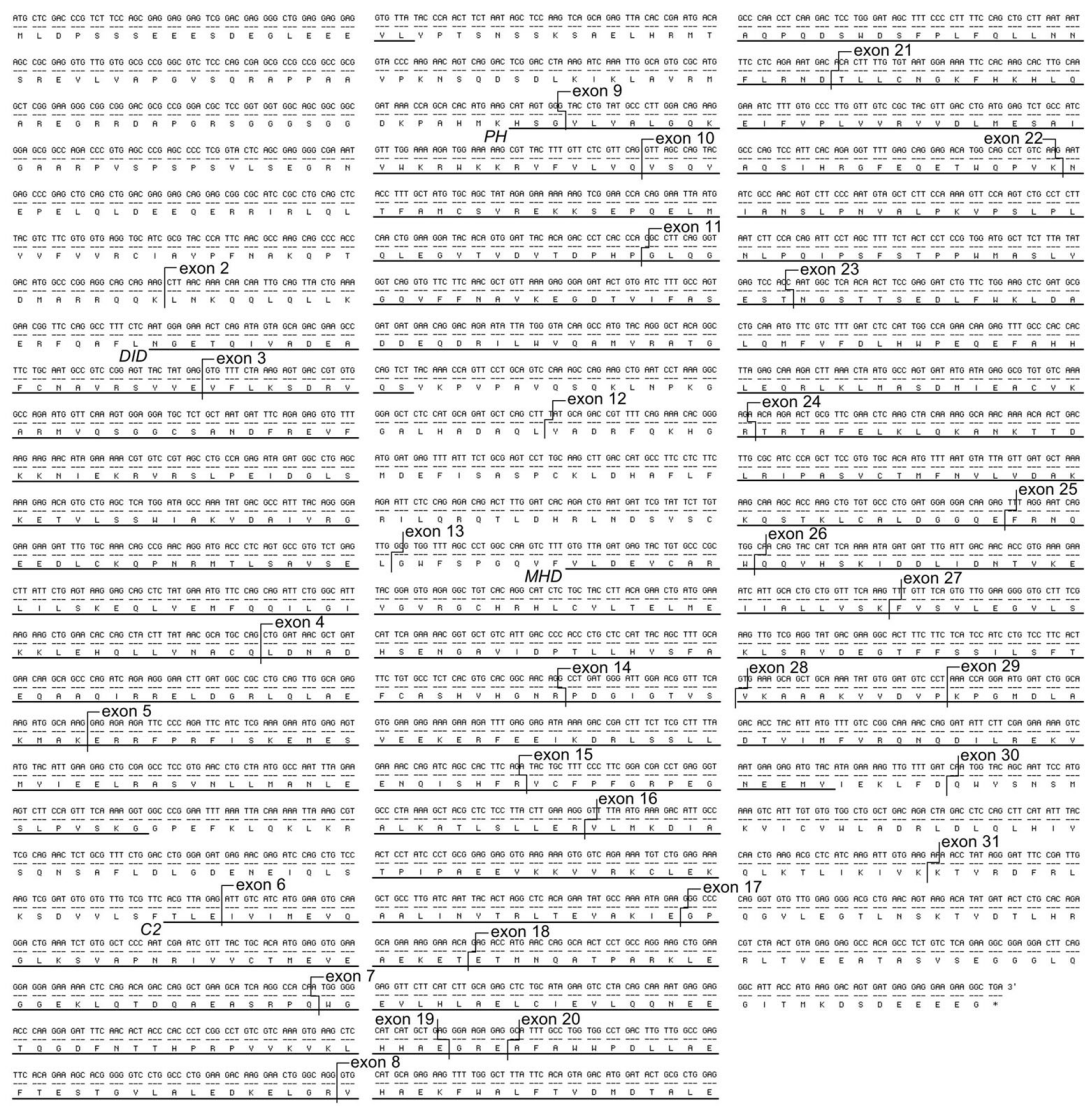
**Nucleotide sequence and deduced amino acid sequence of mouse CAPS2**. The p150^*Glued*^/dynactin 1 interacting domain (DID), C2 domain, PH-like domain, and Munc13-1 homologous domain (MHD) are underlined: aa 134-331, 369–452, 494–596, and 677–1206, respectively.

Using cDNA probes comprising the sequence of both exons 4 and 5 of CAPS2, we screened a P6 mouse cerebellar cDNA library for splicing variants of CAPS2. In addition to the original clone designated CAPS2a [DDBJ: AB098623], which was identical to the one that we previously reported [6,14], we isolated five additional variants, probably derived from full-length transcripts, designated CAPS2b [DDBJ: AB291947], 2c [DDBJ: AB291948], 2d [DDBJ: AB291949], 2e [DDBJ: AB291950], and 2f [DDBJ: AB291946]. Sequence analyses against genome databases showed that these sequences could be produced by the different usage of the 31 exons of the CAPS2 gene (Figure 1). All splicing acceptor and donor sequences of these clones were consistent with the AG/GT rule.

**Figure 2:**
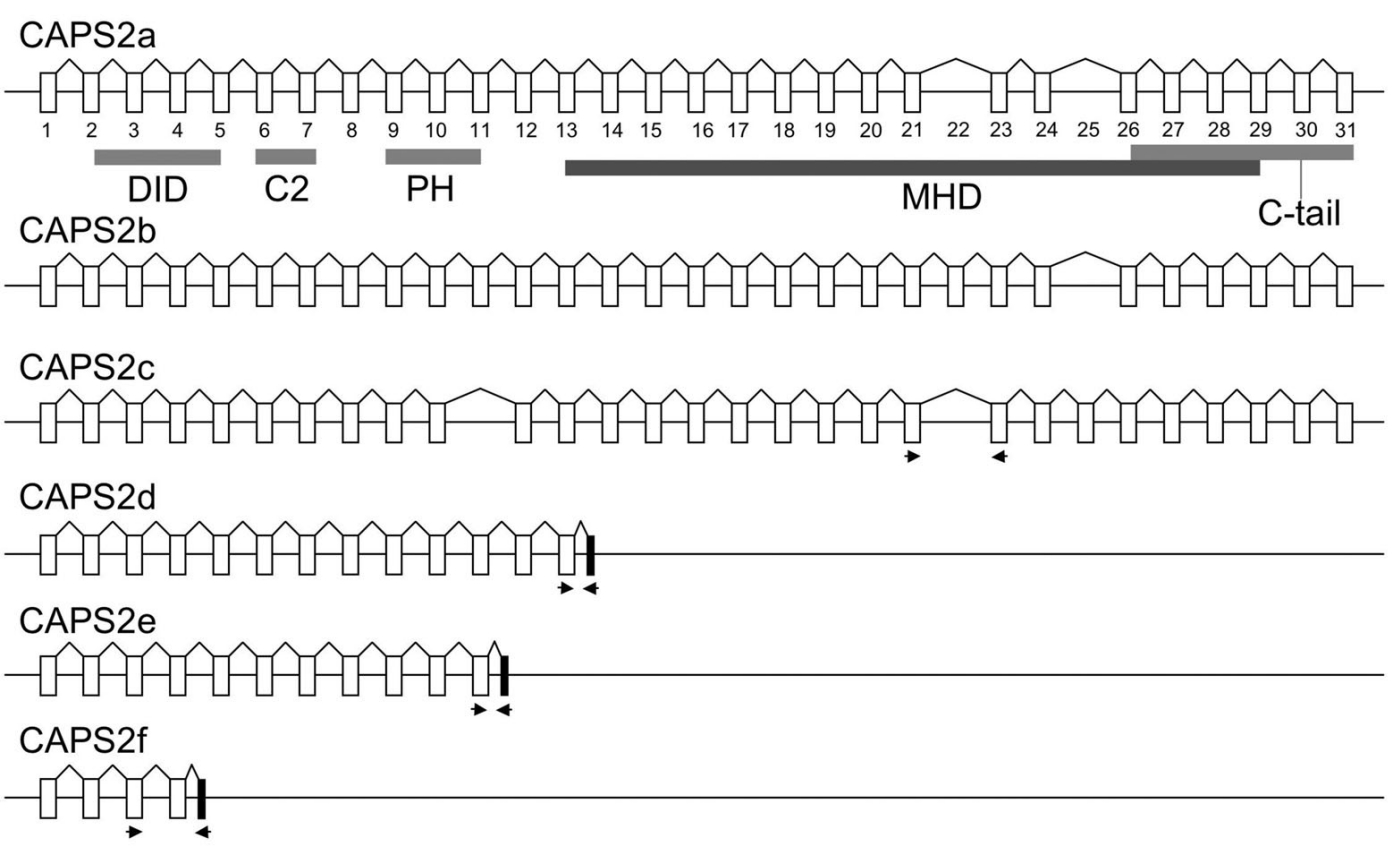
**Exon and intron structures of CAPS2 splice variants**. Alternative splicing to generate CAPS2a, 2b, 2c, 2d, 2e, and 2f cDNAs. Represented here are the p150^*Glued*^/dynactin 1 interacting domain (DID), C2 domain, PH-like domain, Munc13-1 homologous domain (MHD), and the C-terminal domain that mediates DCV binding (C-tail) [7]. The positions of the primers used in the experiments shown in Figures 3B and C are indicated by arrows. Exons used by only one isoform are shown in black rectangles.

The full-length CAPS2 protein consists of the following functional domains, as shown in Figure 2: a dynactin 1-interacting domain (DID), a C2 domain, a pleckstrin homology (PH) domain and a Munc13-1-homologous domain (MHD). CAPS2b lacks only exon 25, which encodes a part of the MHD. CAPS2a lacks two exons, 22 and 25, which encode part of the MHD sequence. CAPS2c lacks two exons, exon 11 encoding a C-terminal part of the PH domain, and exon 22. In contrast to these three long isoforms (CAPS2a-c), CAPS2d, 2e and 2f have large C-terminal large truncations from exon 14, 12, and 5, respectively; thus, these isoforms have neither the MHD nor the C-tail domain, which mediates the association of CAPS with DCVs [7]. These three short isoforms (CAPS2d-f) have an additional unique exon at their 3' end. The shortest isoform, CAPS2f, has only the first four authentic exons and none of the functional domains identified so far.

**Figure 3:**
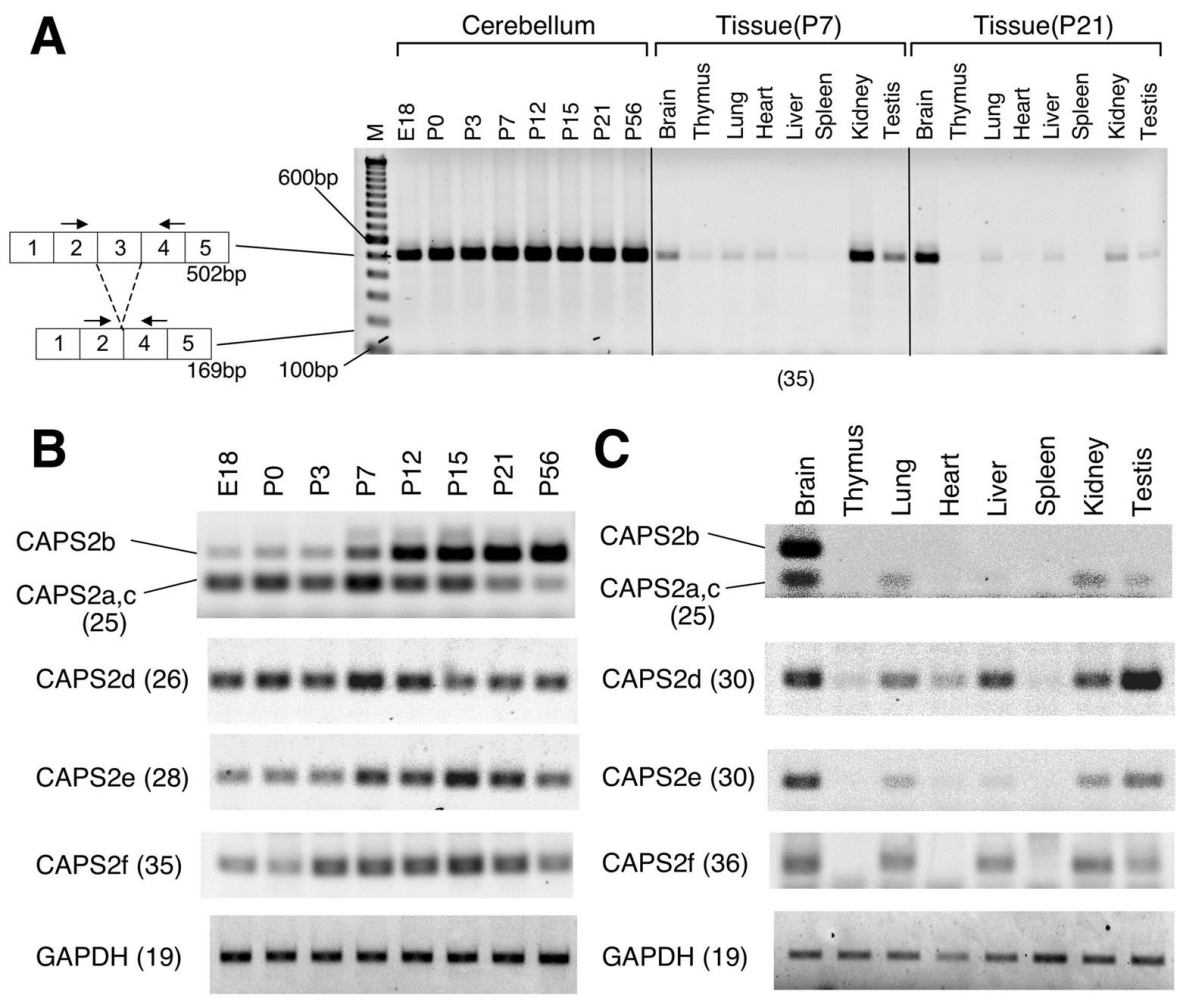
**Developmental expression pattern in cerebellum and tissue distribution of CAPS2 splice variants**. (A) Exon 3-skipping in normal mice. RT-PCR analysis of CAPS2 mRNA in total RNA from developing mouse cerebella (P0, P3, P7, P12, P21, and P56) and various tissues (P7 and P21). An aberrant alternatively spliced CAPS2 mRNA that lacks exon 3 was previously observed in some autistic patients [12]. A single major band (502 bp) is apparent in all samples in which CAPS2 is expressed. A smaller band (169 bp) that would indicate skipping of exon 3 was not detected. The primers used are indicated on the corresponding exons by arrows. Lane M, 100-bp ladder molecular size marker (from 100 bp at the bottom to 1500 bp at the top). (B) The expression profile of CAPS2 isoforms in developing mouse cerebella at E18, P0, P3, P7, P12, P15, P21, and P56 was analyzed by RT-PCR. (C) The tissue distribution of CAPS2 isoform expression in P21 mice was analyzed by RT-PCR. Glyceraldehyde-3-phosphate dehydrogenase (GAPDH) was used as a control. The numbers of PCR cycles are shown in parenthesis.

All isoforms (CAPS2a-f) have exon 3, which contains the domain interacting with p150^*Glued *^[12]. The mouse CAPS2 exon 3 coding sequence is identical to the human CAPS2 exon 3 coding sequence, which is absent in some autistic patients. As shown in Figure 3A, no exon 3 skipping was observed during the development of the cerebellum, from E18 to P56, provided there were sufficient PCR cycles. Moreover, no exon 3 skipping was observed in whole brain, thymus, lung, heart, liver, spleen, kidney, or testis from P7 or P21 mice (Figure 3A). These results suggest that exon 3 skipping does not normally occur in mouse CAPS2.

We examined the patterns of CAPS2 splicing during cerebellar development, by RT-PCR using specific primer sets for each isoform. CAPS2b is the only isoform that contains exon 22. Therefore, RT-PCR with a primer pair spanning exon 21-exon 23 resulted in a longer band (267 bp) for CAPS2b in addition to a shorter band (147 bp) for CAPS2a and 2c. Interestingly, there was a peak in the expression of CAPS2a and/or 2c at P7, whereas CAPS2b was up-regulated throughout development (Figure 3B). The expression of CAPS2d reached a peak at P7 and decreased thereafter, whereas that of both CAPS2e and 2f reached a peak between P12 and P21.

Among the eight mouse tissues tested, the brain, lung, kidney, and testis showed expression of all isoforms except for CAPS2b (Figure 3C). Interestingly, expression of CAPS2b was detectable only in the brain. Among these isoforms, the expression level of CAPS2f was the lowest as shown by its requiring the highest cycle number of PCR cycles to detect it (numerals within the parenthesis of Figure 3B, C).

**Figure 4:**
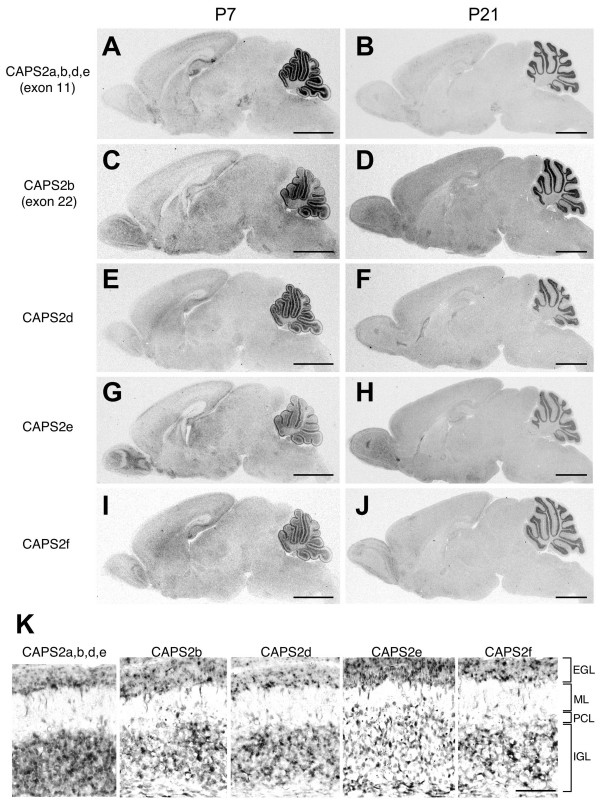
**Expression of CAPS2 splicing variants mRNA in P7 and P21 mouse brains**. (A-J) *In situ *hybridization analysis of the mRNA distribution of CAPS2 isoforms in parasagittal brain sections. (A and B) Exon 11 was used as a probe for CAPS2a, 2b, 2d, and 2e isoforms. (C and D) Exon 22 was used as a probe for the CAPS2b isoform. (E and F) The CAPS2d-specific exon (black rectangle in Figure 2) was used as a probe for the CAPS2d isoform. (G and H) The CAPS2e-specific exon (black rectangle in Figure 2) was used as a probe for the CAPS2e isoform. (I and J) The CAPS2f-specific exon (black rectangle in Figure 2) was used as a probe for the CAPS2f isoform. Scale bar = 2 mm. (K) *In situ *hybridization analysis of the mRNA distribution of CAPS2 isoforms in the P7 cerebellar cortex. EGL, external granule layer; ML, molecular layer; PCL, Purkinje cell layer; IGL, internal granule layer. Scale bar = 50 μm.

We next examined the cellular distribution of CAPS2 isoform mRNAs in P7 and P21 mouse brains by *in situ *hybridization analyses. Exon 11, which contains a part of the PH-domain coding sequence, is skipped in CAPS2c, but is included in CAPS2a, 2b, 2d, and 2e (Figure 2). The probe for exon 11 showed expression in the hippocampal formation, neocortex, midbrain, and cerebellar granule cells (Figures 4A and 4B). Exon 22, which contains a part of the MHD coding sequence, is skipped in CAPS2a and 2c, but is included in CAPS2b (Figure 2). The probe for exon 22 showed an expression pattern with the most prominent level in cerebellar granule cells (Figures 4C and 4D). Expression of CAPS2d, 2e and 2f was also observed mainly in cerebellar granule cells (Figures 4E–J). Moreover, there was no apparent difference in the expression patterns of these isoforms in the P7 cerebellar cortex (granule cells in the external and internal granule layers and migrating granule cells in the molecular layer), as shown in Figure 4K.

**Figure 5:**
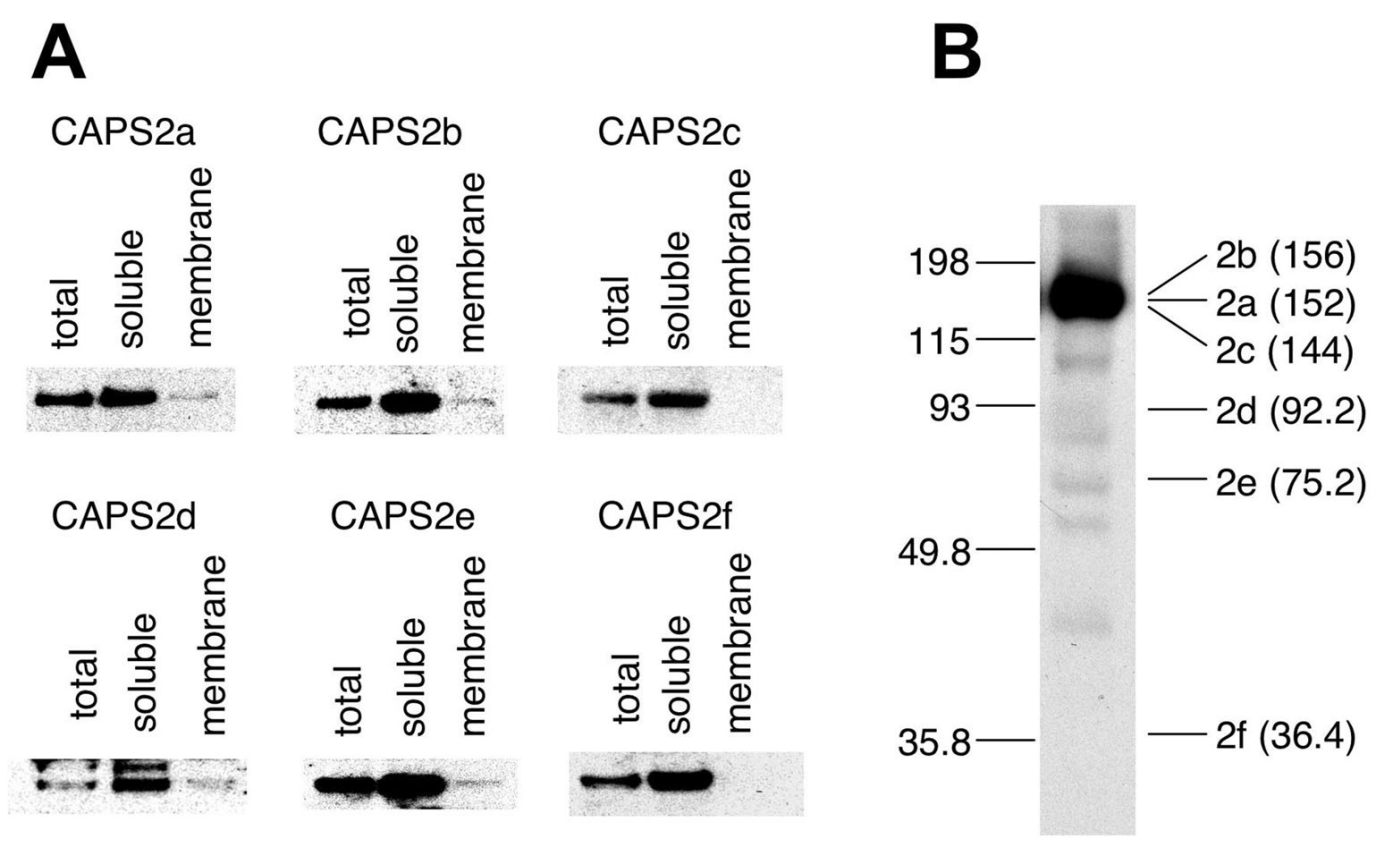
**The subcellular distribution and expression level of CAPS2 splice variants**. (A) C-terminal HA-tagged CAPS2 isoforms were exogenously expressed in PC12 cells. Membrane and soluble fractions were separated as described in the Experimental procedures section. An equal amount of total, soluble and membrane fractions were subjected to SDS-PAGE, followed by immunoblotting with an anti-HA antibody. (B) The expression profile of CAPS2 isoforms in mouse cerebella at P56 was analyzed by Western blotting. The position and size (kDa) of molecular weight markers are indicated on the left. The deduced molecular weight (kDa) of each isoform is indicated in parentheses on the right.

CAPS family proteins are essentially soluble proteins, but a fraction of them is capable of being associated with membrane [2,5]. Therefore, we next examined the subcellular fractionation patterns of these splicing variant proteins, exogenously expressed in PC12 cells. All isoforms were stably expressed in transfected PC12 cells (Figure 5A). CAPS2 isoforms containing an intact PH domain (CAPS2a, 2b, 2d, and 2e) were recovered from the membrane fraction, although a major portion of each isoform was included in the soluble fraction. On the other hand, CAPS2 isoforms lacking a part of the PH domain (CAPS2c) or the entire PH domain (CAPS2f) were not detectable in the membrane fraction, suggesting that the PH domain has a role in the membrane association of CAPS2 protein, as previously shown [7].

We next examined the expression of the endogenous proteins produced from these splice isoform mRNAs in the mouse brain. Immunoreactive bands corresponding to short isoforms (CAPS2d-f) were observed at low to undetectable protein levels in the mouse cerebellum (Figure 5B). In addition, the previous report showed that in non-neural tissue such as pituitary, lung, pancreas and kidney, only a 150 kDa band immunoreactive for CAPS2 was detected [15]. At the mRNA level, more reaction cycles and time were needed for the amplification of transcripts encoding the short isoforms by RT-PCR and for the colorimetric detection of transcripts by *in situ *hybridization, respectively, indicating that the expression levels of the genes encoding the short isoforms are very low. These results indicate that the function of CAPS2 *in vivo *is apparently due to the long isoforms (CAPS2a-c) rather than the short isoforms (CAPS2d-f). However, the functional role(s) of the short isoforms remain to be studied.

**Figure 6:**
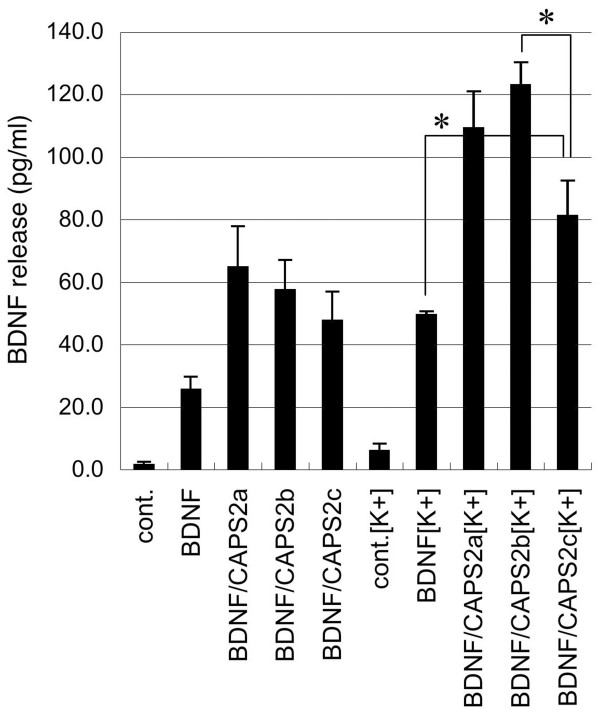
**The functional differences between CAPS2 splice variants**. PC12 cells were transfected with CAPS2 constructs together with a BDNF expression vector. The concentrations of BDNF (pg/ml) in culture media of cells with or without 50 mM KCl stimulation were analyzed by ELISA. PC12 cells express CAPS1, another CAPS family protein, and thus showed increased levels of BDNF release in the presence of high K concentrations [6]. Our recent study also showed that overexpression of CAPS2 not only promotes activity-dependent, regulated release of BDNF, but also slightly increases the basal, constitutive release of BDNF [12]. Data points represent the averages of values obtained from four independent experiments. The error bars indicate the SD. *P < 0.05, by Student's *t *test. The differences between the BDNF releasing activities of three isoforms (CAPS2a, b, and c) after 50 mM KCl stimulation were shown to be significant by one-way ANOVA analysis (*P *< 0.05).

To analyze functional differences between splicing variants, we carried out a BDNF secretion assay (Figure 6). To investigate the effect of these isoforms on BDNF releasing activity, we exogenously expressed the three long isoforms (CAPS2a-c) together with BDNF in PC12 cells. Short isoforms (CAPS2d-f) were not analyzed because of their large C-terminal truncation (Figure 2) and very low expression levels in mouse brain (Figure 5B). As shown in Figure 6, the levels of BDNF released into the culture medium in response to high-KCl stimulation were increased approximately two-fold by the expression of CAPS2a or 2b compared with cells without exogenous CAPS2 expression. CAPS2c-expressing cells showed lower BDNF-releasing activity than CAPS2a- or 2b-expressing cells; however, CAPS2c-expressing cells also showed an increase in BDNF release in response to high-KCl stimulation (Figure 6). Moreover, there was no significant difference in basal BDNF release between CAPS2c-expressing cells and cells expressing either of the other two isoforms (CAPS2a and 2b). There was a partial deletion of the PH domain in CAPS2c (Figure 2). The partial PH domain of PLC-gamma 1 is known to interact with a complementary partial PH-like domain of another protein and elicit its function [16]. Therefore, it is possible that CAPS2c has some BDNF-releasing activity, although its activity is lower than that of intact PH domain-containing isoforms.

Interestingly, expression of the exon 22-containing isoform was up-regulated in the developing mouse cerebellum, whereas exon 22-skipped isoforms were expressed transiently, peaking at P7. Moreover, the exon 22-containing isoform showed higher BDNF-releasing activity than other long isoforms (Figure 3B). On the other hand, as described in our previous report, CAPS2 immunoreactivities are detected in both the soma and axon of cerebellar granule cells at P7, but almost exclusively in the axon at P21 [6]. These results suggest that the exon 22 splicing patterns are related to high releasing activity and axonal localization in cerebellar granule cells. CAPS2-mediated regulation of neurotrophin actions in terms of their releasing dose and subcellular localization may be important for normal cerebellar development.

The splicing of exon 22 is interesting for reasons other than the developmental differences in expression; that is, the exon 22-containing isoform was detectable only in the brain, whereas exon 22-skipped isoforms were widely expressed in various tissues. Exon 22 encodes part of the MHD, specifically a proline-rich domain (8 prolines within 40 residues). Proline-rich motifs occurred often in many protein-protein interaction sites, including one recognized by the Src homology 3 (SH3) domain. Our preliminary screening of protein candidates that interact with the exon 22-encoded region, by yeast two-hybrid, isolated five candidates; however, none of these had an SH3 domain (data not shown). Future analyses of CAPS2 proline-rich domain-interacting proteins will clarify the significance of the alternative splicing of exon 22.

## Conclusion

We explored the alternative splicing diversity of CAPS2 and identified six CAPS2 isoforms. The alternative splicing of exon 22 (presence or absence) was associated with a difference in tissue distribution and a developmental expression pattern. Our study showed that an intact PH domain is required for the association of CAPS2 with the membrane and efficient BDNF release activity. On the other hand, exon 3-skipping, found in autistic patients, was not observed in the mouse tissues tested.

## Methods

### Animals

All experimental protocols were approved by the RIKEN Institutional Animal Care and Use Committee.

### Cloning of mouse CAPS2 splicing variants

DNA containing exons 4 and 5 of CAPS2 was used as a probe to clone mouse CAPS2 splicing variant cDNAs. Approximately 1 × 10^6 ^plaques of a cDNA library constructed from P6 cerebella of C57BL/6j mice (a kind gift from Dr. M. E. Hatten, Rockefeller University, New York, NY) were screened using a ^32^P-labeled probe. Sequencing was performed in both directions by the dideoxy chain termination method using [α-^32^P] dCTP and the BcaBest dideoxy sequencing kit (Takara Bio, Otsu, Shiga, Japan).

### RT-PCR analysis

A series of first-strand cDNAs was produced by reverse-transcription (RT) from 20 ng of total RNA derived from ICR mice using an oligo-dT primer. The cycling conditions were as follows: denaturing at 94°C for 3 min, amplification by cycles of 95°C (30 s), 60°C (30 s), and 72°C (1 min), and extension at 72°C for 5 min. Primers were as follows. Mouse CAPS2 exon 21–23 forward primer: 5'-GACCTGATGGAGTCTGC-3'; exon 21–23 reverse primer: 5'-CCAATGGAGATCAAAGACGAAC-3' (CAPS2b product: 267 bp; CAPS2a, c product: 147 bp); CAPS2d forward primer: 5'-GCTGTCATTGACCCCAC-3'; CAPS2d reverse primer: 5'-GATGAAACAACCCCAGTCAC-3' (CAPS2d PCR product: 220 bp); CAPS2e forward primer: 5'-CAACGCTGTTAAAGAGGGAG-3'; CAPS2e reverse primer: 5'-GCTTCAATTTTGTGTTTTATTAAGGTTATTATTTGATAATC-3' (CAPS2e PCR product: 240 bp); CAPS2f forward primer: 5'-CTGAGTAAGGAGCAGCTC-3'; CAPS2f reverse primer: 5'-GGCCAAGGCAGAAAGGT-3' (CAPS2f PCR product: 225 bp). RT-PCR of mouse glyceraldehyde-3-phosphate dehydrogenase (GAPDH) with the primers 5'-GCCATCAACGACCCCTTCATTGACCTC-3' (forward primer) and 5'-GCCATGTAGGCCATGAGGTCCACCAC-3' (reverse primer) was used as an internal control.

### *In situ *hybridization

DNA sequences corresponding to various exons of CAPS2 (exon 11, exon 22, CAPS2d-specific exon, CAPS2e-specific exon, and CAPS2f-specific exon) were used as templates to prepare digoxigenin-labeled antisense riboprobes using a digoxigenin-dUTP labeling kit (Roche Diagnostics, Indianapolis, IN, USA). Eight μm sections of 4% paraformaldehyde-fixed, paraffin-embedded P7 and P21 mouse brains were deparaffinized and treated with a proteinase K solution (10 ng/μl in PBS; Invitrogen, Carlsbad, CA, USA) at room temperature for 15 min. After acetylation, sections were incubated in a hybridization buffer containing 500 ng/μl digoxigenin-labeled riboprobes, at 60°C overnight, in a humidified chamber. Hybridized sections were washed by successive immersion in 2 × SSC, 50% formamide (at 60°C for 20 min, twice), TNE (1 mM EDTA, 0.5 M NaCl, 10 mM Tris-HCl, pH 8.0, at 37°C for 10 min), TNE containing 20 ng/μl RNase A (at 37°C for 30 min), 2 × SSC (at room temperature for 10 min, twice), and 0.2× SSC (at 60°C for 30 min, twice). The hybridization signals were detected using a digoxigenin detection kit (Roche diagnostics).

### Subcellular fractionation

Subcellular fractionation was performed as described previously [17]. Mouse CAPS2 cDNAs were subcloned into the pEF-BOS vector containing the EF-1α promoter [18] and a C-terminal hemagglutinin (HA) tag to create pEF-BOS-CAPS2-HA. PC12 cells (1 × 10^6 ^cells) transfected with pEF-BOS-CAPS2-HA constructs, using LIPOFECTAMINE 2000 Reagent (Invitrogen) according to the manufacturer's instructions, were harvested 1 day after transfection and suspended in 1 ml of a homogenization buffer (10 mM Tris-HCl (pH 7.5), 0.32 M sucrose, 1 mM EDTA and a 1× cocktail of protease inhibitors) and passed five times through a 26-gauge needle. The homogenate was centrifuged at 1,000 × *g *for 10 min at 4°C. The supernatant was further centrifuged at 100,000 × *g *for 1 h at 4°C and the resulting supernatant was saved as the cytosolic fraction. The pellet was resuspended in 1 ml of homogenization buffer (crude membrane fraction). Protein concentrations in each fraction were quantified using bicinchoninic acid (BCA protein assay; Pierce, Rockford, IL, USA), and equal amounts of protein were separated by SDS-PAGE and subjected to Western blotting.

### Preparation of subcellular fractions

P56 C57BL/6J male mice were sacrificed after anesthesia with diethyl ether. Mouse cerebella were dissected and homogenized in ice-cold homogenization buffer (50 mM HEPES [pH 7.4], 150 mM NaCl, 10% Glycerol, 1% Triton X-100, 5 mM EDTA, protease inhibitor cocktail) using a glass/Teflon homogenizer. Homogenates were centrifuged at 20,000 × *g *for 10 min at 4°C. The protein concentrations of lysates were quantified using a BCA protein assay. Ten μg of protein was separated by SDS-PAGE and subjected to Western blotting.

### Antibodies

Guinea pig polyclonal anti-CAPS2 antibody raised against the GST-tagged CAPS2 (aa 18–89) [6] and rat monoclonal anti-HA antibody (cat. no. 1867423, Boehringer Mannheim, Indianapolis, IN, USA) were used for Western blotting (used at dilution of 1:10,000 and 1:1,000, respectively).

### Secretion assay

Mouse BDNF cDNA was subcloned into the pEF4/Myc-His plasmid vector containing the EF-1α promoter (Invitrogen) to create pEF4/Myc-His-BDNF. Mouse CAPS2 cDNA was subcloned into the pcDNA3 plasmid vector containing the CMV promoter (Invitrogen) to create pcDNA3-CAPS2. Transfections were carried out in 24-well plates, using LIPOFECTAMINE 2000 Reagent (Invitrogen) as described previously [6]. For these experiments we applied LIPOFECTAMINE complexed with 1 μg of pcDNA3-CAPS2 and 0.05 μg of pEF4/Myc-His-BDNF plasmid DNAs to PC12 cells. Following 5 h of incubation with the LIPOFECTAMINE and plasmid DNA complexes, cells were washed and grown in normal medium. At 24 h after transfection, PC12 cells were incubated in fresh assay medium (DMEM containing 0.2% BSA) for 10 min. Two different DMEMs were used: standard DMEM (11965; Invitrogen) and high-KCl DMEM (50 mM KCl, 65 mM NaCl based on 11965; specially ordered; Invitrogen). Both DMEMs were fully equilibrated in a 5% CO_2 _atmosphere at 37°C before application. Culture media were collected after control and high-KCl stimulation assays, and the neurotrophin concentration (pg/ml) was measured using the BDNF Emax ImmunoAssay System (Promega, Madison, WI, USA) according to the manufacturer's instructions.

### Statistical analysis

Statistical analysis was performed using Statcel software (OMS, Saitama, Japan). Data were compared using the Student's *t *test or the Mann-Whitney *U *test according to unequal or equal variance. Comparisons between groups of splicing variants were made using one-way ANOVA. A *P *value of <0.05 was considered statistically significant.

## Authors' contributions

TS made contributions to the conception and design of the study, the acquisition of data, and the analysis and interpretation of data, and drafted the manuscript. MW also made a contribution to the acquisition of data. TF conceived of the study and helped to draft the manuscript. All authors read and approved the final manuscript.
